# An Optimization Method of Deep Transfer Learning for Vegetation Segmentation under Rainy and Dry Season Differences in a Dry Thermal Valley

**DOI:** 10.3390/plants12193383

**Published:** 2023-09-25

**Authors:** Yayong Chen, Beibei Zhou, Dapeng Ye, Lei Cui, Lei Feng, Xiaojie Han

**Affiliations:** 1 College of Mechanical and Electrical Engineering, Fujian Agriculture and Forestry University, Fuzhou 350012, China; yysuper@fafu.edu.cn; 2 Fujian Key Laboratory of Agricultural Information Sensoring Technology, Fuzhou 350012, China; 3 State Key Laboratory of Eco-Hydraulics in Northwest Arid Region of China, Xi’an University of Technology, Xi’an 710048, China; 4 China Renewable Energy Engineering Institute, Beijing 100032, China; zan350639414@163.com; 5 Central South Survey and Design Institute Group Co., Ltd., Changsha 410014, China; pingchen43982@163.com; 6 China Electric Construction Group Beijing Survey and Design Institute Co., Beijing 100024, China; longhao69563082@163.com

**Keywords:** vegetation in field, deep learning optimization, dry-hot valley environment, migration training, best migration learning conditions

## Abstract

Deep learning networks might require re-training for different datasets, consuming significant manual labeling and training time. Transfer learning uses little new data and training time to enable pre-trained network segmentation in relevant scenarios (e.g., different vegetation images in rainy and dry seasons); however, existing transfer learning methods lack systematicity and controllability. So, an MTPI method (Maximum Transfer Potential Index method) was proposed to find the optimal conditions in data and feature quantity for transfer learning (MTPI conditions) in this study. The four pre-trained deep networks (Seg-Net (Semantic Segmentation Networks), FCN (Fully Convolutional Networks), Mobile net v2, and Res-Net 50 (Residual Network)) using the rainy season dataset showed that Res-Net 50 had the best accuracy with 93.58% and an WIoU (weight Intersection over Union) of 88.14%, most worthy to transfer training in vegetation segmentation. By obtaining each layer’s TPI performance (Transfer Potential Index) of the pre-trained Res-Net 50, the MTPI method results show that the 1000-TDS and 37-TP were estimated as the best training speed with the smallest dataset and a small error risk. The MTPI transfer learning results show 91.56% accuracy and 84.86% WIoU with 90% new dataset reduction and 90% iteration reduction, which is informative for deep networks in segmentation tasks between complex vegetation scenes.

## 1. Introduction

Vegetation segmentation is an important basic operation of identifying and extracting vegetation regions in satellite or aerial images using computer vision techniques [1]. Obtaining plant information in the actual environment is of great significance in the fields of agricultural information monitoring [2], environmental data investigation [3], soil and water conservation [4] and ecological information statistics [5]. Currently, an efficient, accurate, and flexible vegetation segmentation processing method is urgently needed to meet the ever-increasing image data, the increasingly meticulous vegetation assessment, and the monitoring requirements [6]. By extracting image features and automatically improving their accuracy, these deep learning methods can greatly simplify the overall workflows and improve the accuracies of the wild vegetation information surveys [7,8]. However, deep learning for vegetation segmentation still faces the problems of dataset labeling and training time cost [9,10]. Thus, many experts and scholars are working on improvements such as enhanced network structures [11,12], more efficient data sources [13], and further optimization existing network features [14].

Network structure enhancements have wide applications in agricultural tasks, e.g., H. Sheng [11], S.Y. Zhao [12], and N. Lin [15] improved deep network feature extraction and enhanced network accuracy by adding attention mechanisms. Under limited computational capacity, the attention structure in deep networks (mimicking the attention of a biological brain) allocates more resources to the most important features while reducing or even filtering out less relevant computational to improv efficiency and accuracy [16]. Similar to attentional structures, LSTM (Long Short-Term Memory) structures, mimicking biological memories [17,18,19], can enhance information delivery and improve the network learning ability [20] because the cumulative updating strategy and forgetting/memory gating methods enhance gradient transfer [21,22]. Additionally, the Pyramid Pooling Module structure [23,24] and self-encoder structure [25,26] can also reduce the vanishing gradient problem and improve the training effectiveness of deep learning [27]. Although structural improvement methods can improve the network capacity, such methods may increase the feature quantity [28], which requires more data and training costs.

In addition to the improvements in network structure, the quality and quantity of data can significantly impact deep learning. V. Balagurusamy [13], B. Sorscher [29], K. Alzhrani [30], and R. P. Kosaraju [31] reported that pruning the dataset could improve the model effectiveness and increase the training speed. S. Yang [32] also pointed out that the dataset pruning is mainly achieved by removing redundant or irrelevant data, which reduces the required training time but may change the data distribution and lead to valid data loss. Additionally, increasing the data size by GAN (Generative Adversarial Networks) is also a common approach, such as training GAN models on existing images data and enlarging the dataset to improve the network accuracy, as proposed by V. Giuffrida [33], F. Zhu [34], Q. Zeng [35], and B. Liu [36]. Of course, data augmentation with traditional image processing methods is also a very meaningful approach to enhance the networks robustness and accuracy, such as methods proposed by G. Geetharamani [37], S. Zhuang [38], and A. Fuentes [39]. Although these dataset extension methods can simplify the manual labeling process, they also increase the computational cost.

The transfer learning introduced by R. Yu [40], M. Zhen [41], and A. O. Agbaje [42] is also a deep network optimization process that has significant advantages in both training time and dataset size. Transfer learning focuses on how to make a pre-trained network applicable to a new relevant task, using only minimal new data and training cost [43]. For example, Y.J. Wang [44], M. Hussain [45], N. Best [46], and J. Chen [14] froze some feature layers of networks and achieved good accuracy with little new data (e.g., 80 [47]). However, most existing transfer learning methods were implemented based on TaE (trial-and-error method), such as N. Best [46] and Y.J. Wang [44], which might still need further improvement in systemic and controllability overall.

Based on the above discussion and analysis, transfer learning is suitable for vegetation segmentation tasks between complex environments in the wild. So, in this study, a vegetation segmentation deep network pre-trained in the rainy season was transferred to the dry season environment using little new data and training time in DTV (Dry Thermal Valley, where there are significant seasonal variations differences). In the pre-training phase, four deep networks, including Seg-Net (Semantic Segmentation Networks), FCN (Fully Convolutional Networks), Mobile-Net v2, and Res-Net 50 (Semantic Segmentation Networks), were constructed and pre-trained with rainy season vegetation data in order to select the best network (Res-Net 50) for subsequent transfer optimization. Thereafter, an MTPI method (Maximum Transfer Potential Index) was proposed to find the optimal transfer conditions, which evaluated and optimized both data and feature volume to accurately migrate the pre-trained network with dry season vegetation data. The main purposes of our study are to reduce the TaE cost, improve the risk controllability of the transfer learning, and supply a reference for vegetation information extraction in the wild, especially in the reduction in datasets and training time for vegetation segmentation multitasking in complex environments.

## 2. Results and Discussions

### 2.1. The Four Pre-Trained Deep Vegetation Segmentation Networks

**Figure 1:**
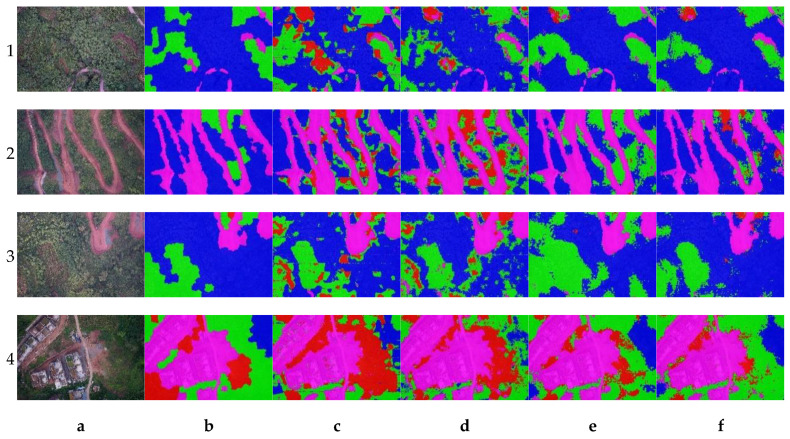
Results of the four pre-trained deep learning networks for the segmentation of four random samples. (**a**). RGB image. (**b**). Reference label mapping. (**c**). Results of a-image processing using Seg-Net. (**d**). Results of a-image processing using FCN. (**e**). Results of a-image processing using Mobile-Net v2. (**f**). Results of a-image processing using Res-net50. No. 1 to 4 represent randomized 4 sample results on Rows 1 to 4.

Figure 1 gives the vegetation segmentation images of four vegetation covers using the four deep learning networks in the wilderness experiment. The segmented regions of trees, shrubs, grasses, and NVA were labeled with blue, red, green, and magenta markers with 20% transparency for each displayed. Column a of each row represents the original RGB image, whereas column b represents the manually labeled result, and columns c, d, e, and f represent the segmentation results obtained using the Seg-Net, FCN, Mobile-Net v2, and Res-Net 50 networks, where the references are the images in column b. In general, these four deep learning networks achieved vegetation segmentation; for example, all four deep networks achieved a good result on the NVA compared with column b while also showing that the NVA segmentation task was a simpler part (features such as color texture were visible and evident to the human eyes). Moreover, the Mobile-Net v2 considered the shrubs more as trees, e.g., Figure 1(1e) (1.e represents the resulting image in row 1 and column e). In Figure 1(2e), the FCN misclassified shrubs as grasses more, which might have been related to the convolution depth of the network, where deeper networks have better resultant performance. In exception, both Mobile-Net v2 and Res-net 50 performed well in the segmentation results of grass and NVA, e.g., Figure 1(1e,1f). But, in shrub regions, Res-net 50 was still significantly better than Mobile-Net v2, e.g., the difference between Figure 1(3e,3f), which may have been related to the performance structure of the Res-net 50. Among these results, the segmentation effect of Res-net 50 was the best among all vegetation species, which may have been related to the residual structure of the network introduced in Res-net 50. This was consistent with the results of A. A. Alf [48] and H. Yu [49].

**Figure 2:**
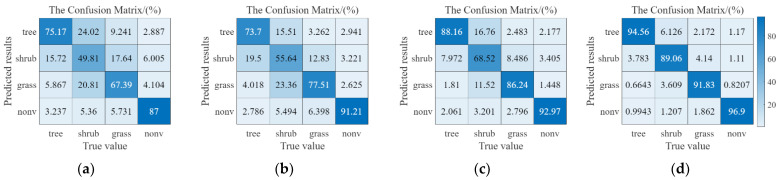
Confusion matrix results of the four pre-trained networks on the ${DA}_{1}$. (**a**). Confusion matrix of Seg-Net. (**b**). Confusion matrix of FCN. (**c**). Confusion matrix of Mobile-Net v2. (**d**). Confusion matrix of Res-net50.

Figure 2 shows the Confusion Matrix results (an overall statistical result of a dataset) for the four pre-trained deep learning networks on the test dataset (${DA}_{1}$ introduced in Section 4.1). The vertical axis is the pixel point output results for each category, whereas the horizontal axis represents the actual category (percentage) within that output category, i.e., the column a on the vertical axis corresponds to the NVA category subdivided into four categories: tree, shrub, grass, and NVA. The diagonal elements of the confusion matrix from the top left to the bottom right reflect the accuracy of the model for each category, with higher values showing better model performances. The results showed that all four deep learning networks achieved some performances on the dataset, with Res-net 50 achieving the highest overall accuracy (94.56, 89.06, 91.83, and 96.90 (%) in Figure 2d, whereas Seg-Net showed the lowest overall accuracy (75.17, 49.81, 67.39, and 87.00 (%) in Figure 2a. Figure 2 showed that Seg-Net had a weak segmentation performed for shrub and grass (Figure 2a, only about 50% and 67%), and FCN was slightly better than that of Seg-Net for grass and shrub (about 10% improvement on grass and shrubs, Figure 2b, whereas the results of Mobile-Net v2 had some degree of accuracy better than Seg-Net and FCN for all four vegetation categories (about 10% improvement than FCN for all categories in Figure 2c; on the other hand, Res-net 50 was better than the three previous network structures in all categories (Figure 2d). These results might have been related to the residual connections structure in Res-net 50, as residual structures could be efficiently learned at deeper levels without being prone to overfitting, which was consistent with previous findings.

Table 1 gives the four pre-trained deep learning networks’ results on the ${DA}_{1}$ from five perspectives (WPA, WRE, WF1, WKs, and WIoU). Among them, WPA refers to the accuracy of pixel segmentation; WRE is the ability of the model to find positive pixels; WF1 evaluates the comprehensive performance of the model; WKs is the Kappa coefficient, being the overall classification accuracy; and WIoU is the overlap of the real regions of the predicted regions. Based on the above five evaluation perspectives, Res-Net 50 was found better overall performance than Seg-Net or FCN, (e.g., the WIoU matrixes in Figure 2a–d. This may have been attributed to the fused residual structure of the Res-Net 50 network with deeper network layers and better vegetation segmentation capabilities. Combining the above example analysis (Figure 1) and the statistical results analysis (Figure 2 and Table 1), the pre-trained Res-net 50 outperformed the other three networks in terms of overall segmentation effect and per-class segmentation effect. This also indicated that Res-net 50 performed well on simple tasks (NVA class segmentation) and complex tasks (grass, shrubs, and trees), both in detail (local results in Figure 2) and overall (Table 1). Therefore, the later work in this paper was discussed using the Res-net 50 network as an example.

### 2.2. Pre-Trained Networks Performance with Rainy and Dry Season Data

Table 2 shows the segmentation results of the pre-trained Res-net 50 on ${DA}_{2,3,4}$ (built in Section 2.2). Comparing the values of ${DA}_{2,3,\;4}$ (e.g., WPA), it could be found that the segmentation effect of ${DA}_{2}$ (0.9352) was better than ${DA}_{3,\;4}$ (0.4382 and 0.1705). The results illustrated that the Res-net 50 pre-trained with the rainy season shady slope data from a DTV still had a strong segmentation effect for rainy season sunny slope data, but no longer fitted for dry season shady or sunny slope data. This might have been because vegetation image characteristics (e.g., color and texture) were influenced by rainy and dry season variability (primarily a moisture factor) in DTV environments, which complemented the image sample image differences in the Section 3.2. In addition, the results of ${DA}_{3}$ were slightly better than ${DA}_{4}$, where WPA, WRE, and WF1 were all higher than ${DA}_{4}$, which might have shown that sunny slopes were more water-scarce than shady slopes in the dry season.

**Figure 3:**
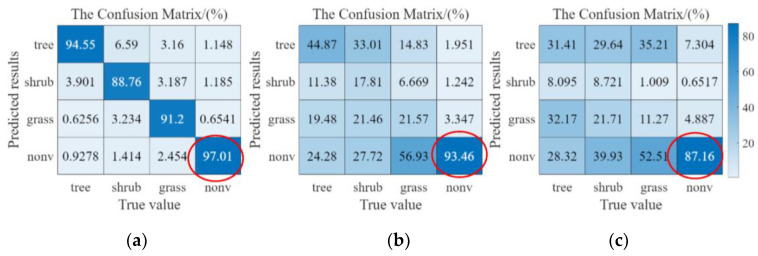
Confusion matrixes results of the pre-trained Res-net 50 on the ${DA}_{2,3,4}$ (**a**). Confusion matrix on the ${DA}_{2}$. (**b**). Confusion matrix on the ${DA}_{3}$. (**c**). Confusion matrix on the ${DA}_{4}$. The red labeling indicates that the pretrained network has high accuracy for NVA classification in all datasets.

The three confusion matrices given in Figure 3 were obtained using the Res-net 50 network pairs pre-trained in Section 2.1 and ${DA}_{2,3,4}$. Comparing the overall results in Figure 3a–c, the pre-trained Res-net 50 still performed well in ${DA}_{2}$ but weakly in ${DA}_{3,4}$ (e.g., the top left to the bottom right values), indicating that a network pre-trained with the rainy shady data still segmented effectively on the rainy sunny data but weakly on dry season data. The confusion matrixes in Figure 3a–c showed that the Res-net 50 network values pre-trained using ${DA}_{2}$ were significantly better than those in ${DA}_{3,4}$ e.g., the top left to the bottom right values of Figure 3a,b, which suggested that the rainy day data were still effective for the four vegetation types on the rainy day side but were weaker for the dry sunny day and rainy day data (for the three vegetation types of trees, shrubs, and grasslands). This also showed that the color, texture, and other characteristic data of the three vegetation types of grass, irrigation, and trees were evidently influenced by the rainy and dry seasons. In addition, the segmentation effects of the NVA in Figure 3a–c (the three results data in red circles) still maintained great accuracy. This might have been because the image features in the NVA were less affected by the rainy and dry seasons factors, and the image features between NVA and vegetated areas were also evident, making the pre-trained deep network achievable acceptable in all datasets.

**Figure 4:**
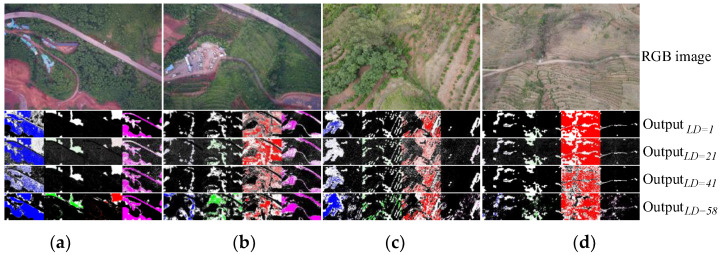
Feature outputs (TPI mapping) of random samples in $D_{1,2,3,4}$ at different layer depths (TP) of the pre-trained res-net 50. (**a**). Random samples in $D_{1}$. (**b**). Random samples in $D_{2}$. (**c**). Random samples in $D_{3}$. (**d**). Random samples in $D_{4}$. (The label image would be resized when the resulting dimension size was different from the label).

Figure 4 showed the feature output (TPI mapping) results of randomly selected samples from ${DA}_{1,2,3,4}$ at different layer depths of the pre-trained network. The color images showed the original RGB samples, and the index mapping showed the feature outputs for different TPs, including four kinds of tree, shrub, grass, and NVA from left to right. Among them, blue, green, red, and magenta regions were, respectively, indicated with strong feature outputs and were consistent with the actual tree, shrub, grass, and NVA targets. The black regions showed that there were neither significant feature regions nor corresponding vegetation regions at the TPs in the pre-trained Res-net 50. White regions meant distinctive features, but without overlapping with corresponding vegetation areas. Therefore, the larger areas of red, green, blue, and magenta regions in the figure indicated that the features of the TP were more evident and more favorable to the segmentation results. The larger areas of the white regions showed the lower overlap between the feature output and the actual vegetation, which was not favorable to segmentation. Therefore, the pre-trained network had a great feature extraction effect and output effect with ${DA}_{1,2}$, such as the regions with evident features and high overlaps for each feature layer in Figure 4a. However, different TPs in the pre-trained network were still extracted from feature regions of a certain range in the $D_{3,4}$, but, still, few regions did not overlap well with the actual regions and were not perfected well in the higher TP, as shown in the grass output in Figure 4d. Therefore, the low TP feature part was left unmodified in the pre-trained network for $D_{3,4}\;$segmentation tasks, and only transfer training was performed in the high TP part for optimization. Such a processing method could probably make the updated features less and the network layers shallower in the training task, which effectively reduced the amount of training data required and enhanced optimization training efficiency.

**Figure 5:**
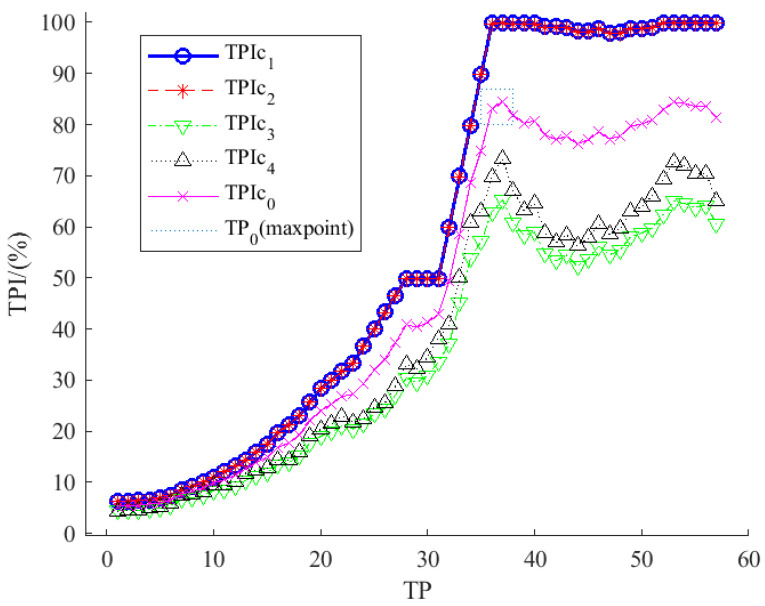
The relationship between TPI curves and the TP in the pre-training network on ${DA}_{1,2,3,4}$. TP means TP, ${TPIc}_{0}$ is the average of ${TPIc}_{1,2,3,4}$, and ${TP}_{0}$ is the max point of ${TPIc}_{0}$.

Figure 5 showed the results of TPIc (TPI curve, conform to Equation (8)) for each LP of the pretraining network in $D_{1,2,3,4}$. Where the *x*-axis was the network TP, and the *y*-axis was the TPI, which meant the transfer potential of the network layer. The general trend in the TPIc showed that the TPIc showed a rapid upward trend and then leveled off or fluctuated with an up-and-down trend as the TP in the pre-trained network increased. This changing scenario might have been determined by the characteristics of the TPI since the feature size factor (the number of features that needed to be trained) for the network transfer was more unfavorable when the TP was small, and, even if the network had a higher potential for accuracy at this point, its overall evaluation index was still small. As the TP elevated, the migrated feature size decreased rapidly, which made the curve rise. When the TP became larger, the feature size was no longer the main influence, and the accuracy influence of transfer began to appear, making the TPIc flatten or fluctuate up and down; this regularity was also responding to the basic characteristics of the TPI. The maximum values of ${TPIc}_{1,2}$ were close to 100%, which meant that the starting point of transfer learning was close to perfect, which was consistent with the earlier conclusion. In addition, the curves showed the results of ${TPIc}_{1}$ ≈ ${TPIc}_{2}$ > ${TPIc}_{3}$ > ${TPIc}_{4}$, which indicated that the pre-trained network had good segmentation effects in the $D_{1,2}$, but poor results in the combination of $D_{3,4}$ ($D_{3}$ was better than $D_{4}$), which corresponded to the starting point of transfer learning, and this feature was also consistent with the test results of the previous pre-trained network. The ${TPIc}_{0}$ (average of ${TPIc}_{1,2,3,4}$) described the potential of the pre-trained networks for $D_{1,2,3,4}$ in the transfer task, whereas the available (near-optimal solution) network depths for $D_{1,2,3,4}$ were obtained by obtaining the maximum value of ${TPIc}_{0}$ (the value determined in this paper was TP_0_ = 37).

### 2.3. Transfer Learning Results in TPI Condition

**Figure 6:**
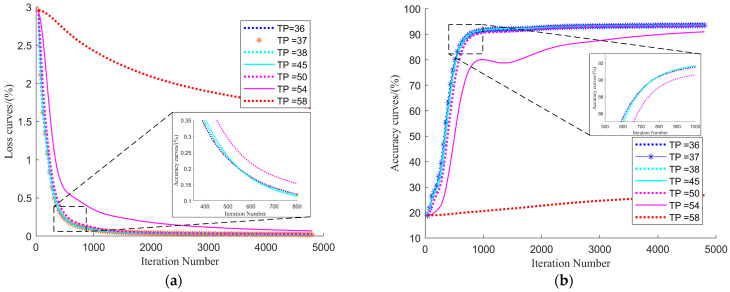
The loss and accuracy trends for the pre-trained network transfer to ${DA}_{3}$, where the TP is around TP = ${TP}_{0}$. (**a**). Loss of transfer. (**b**). Accuracy of transfer. (Curves of layer depth = 57 is also equal to the layer depth = −1 because the maximum network layer of Res-net 50 used in this study is 57.).

Figure 6 shows the loss and accuracy trends for the pre-trained network transfer to ${DA}_{3}$, where the TP was around $TP={TP}_{0}$ (${TP}_{0}$, the result of Section 2.2), with a TDS of 1000 and a maximum iteration number of 1000. As shown in Figure 6a, the network with transfer learning significantly outperformed the TP = 0 in learning efficiency (iterations needed for convergence). This might have been because that the transfer learning was an optimization process based on pre-trained networks since most the network parameters had clear effects in similar tasks (e.g., Figure 6. in Section 2.2) and did not have to be perfected again. Comparing the loss curves for different TPs in Figure 6a, it was shown that the larger the TP was the faster the training speed would be. This might have been because the larger the TP was the fewer layers and the smaller the number of features would be perfected, which made stabilization easier. However, Figure 6b showed that the higher the TP the lower final accuracy was, which was probably due to the less optimized features not being sufficient for the segmentation task. Such results also showed that the TPI parameters proposed in this paper were informative in balancing the learning time cost and the final accuracy in transfer training.

**Figure 7:**
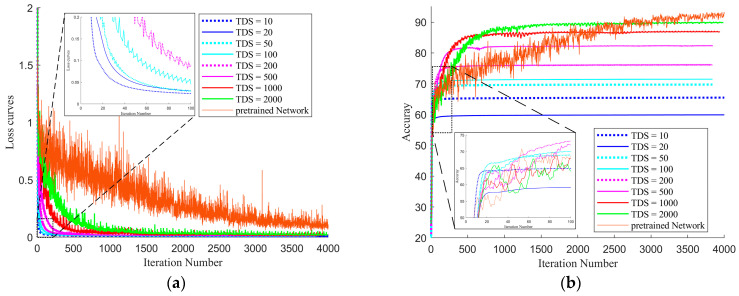
Transfer training process and results for different transfer-set sizes. (**a**). Loss curves in transfer training. (**b**). Accuracy curves in transfer training.

Figure 7 shows the effect of transfer training for pre-trained networks with different dataset sizes and the same TP (=TP_0_). As the figure shows, the larger the dataset used the larger iterations needed, which was a proven result in deep learning, and such regularity was similar to the process of transfer learning. In addition, when TDS received larger values, the results were better, and, when TDS was close to ${TDS}_{0}$ (MTPI condition, 1000 in this paper), the transfer results were close to the pre-trained network effect. However, the actual learning process of deep networks and the separations of datasets in different tasks were highly stochastic, and a certain amount of data margin needed to be left to ensure the actual effect of transfer learning.

**Figure 8:**
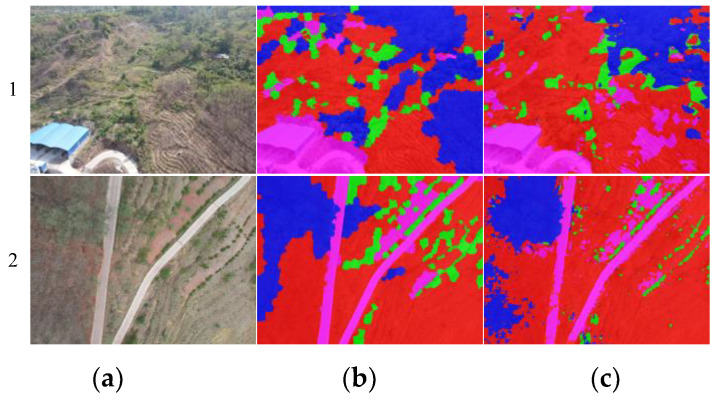
Sample effect of network segmentation after transfer training. (**a**). The original RGB image. (**b**). The manually labeled image. (**c**). The result segmented by the transferred (MTPI transfer) network. Row 1 result is the randomized sample results in D3. Row 2 result is the randomized sample results in D4.

Figure 8 shows two random samples of the transfer learning results of the pre-trained network under MTPI conditions, where the first row was a random sample of $D_{3}$ after transfer, and row 2 was a random sample of $D_{4}$. Among them, column a was the original RGB image, column b was the manually labeled image, and column c was the result segmented by the transferred network. The segmented regions of trees, shrubs, grass, and NVA were labeled with blue, red, green, and magenta markers with 20% transparency, respectively. From the segmentation results shown in Figure 8, the networks after transfer learning could segment vegetation in the rainy and dry seasons of DTV to an extent. It also showed that transfer learning could be effectively implemented in MTPI conditions, which could be quickly and efficiently perfected for the adaptability of deep networks in similar tasks. Although the segmentation results might not have performed perfectly, such as the shrub segmentation results in a.1, this was a transfer result achieved with about 10% iterations and 10% data size, which were acceptable results under the combined cost and accuracy condition. It also showed that transfer learning under TPI conditions could significantly reduce the manually labeled data and the training cost required by the network to improve the capability for a new task.

**Figure 9:**
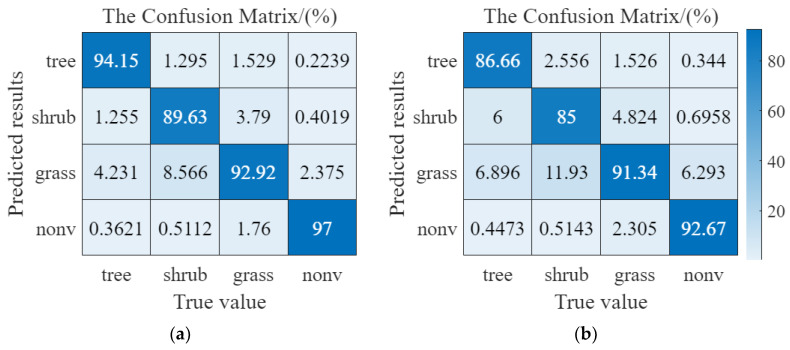
Confusion matrix of test results for two migrated training networks. (**a**). Confusion matrix of network transfer to ${DA}_{3}$. (**b**). Confusion matrix of network transfer to ${DA}_{4}$.

Figure 9 shows the confusion matrixes results of the two deep learning networks after transfer learning for the ${DA}_{3,4}$, respectively. The results showed that the deep network of transfer learning achieved acceptable accuracy on both datasets, whereas the results in ${DA}_{3}$ were better than those in ${DA}_{4}$, for example, in the accuracy of shrubs. The probable reason was that the pre-trained network performed inherently better on ${DA}_{3}$ than ${DA}_{4}$, and the transfer training results at the same TPI conditions also had persistence. In Figure 9b, more shrubs were misclassified as grass (about 12%), which could have possibly been because the differences in image characteristics between trees and shrubs were less than other groups (four groups in total: trees, shrubs, grasses, and NVA), which could also have been related to the distribution of water between vegetation on the sunny slopes under DTV conditions. The five results (WPA, WRE, WF1, WKs, and WIoU) of ${DA}_{3}$ were 93.19%, 93.42%, 93.18%, 86.90%, and 87.69%, whereas they were 89.92%, 88.92%, 89.90%, 85.70%, and 82.02% in ${DA}_{4}$. In addition, the TPI condition was a comprehensive result (approximate best solution) evaluated with efficiency and accuracy in mind, and the actual application may require further optimization based on the TPI condition, according to the complexity of the task, the complexity of the data, and the actual training results.

To verify the advantages of the MTPI condition proposed in this paper, three methods of deep learning transfer training of three types (including 10 studies) were referenced for comparative discussion. Their transfer learning process could be transformed to TPI conditions for discussion, for example, M. Zhen [41], A.O. Agbaje [42], Y. J. Wang [44], A. Abbas [50], and J. Chen [14] froze all layers except for the output layer classification for transfer; thus, this type of method (method one) was transformed to TPI conditions equivalent to the TP = −1 condition. In contrast to the above, method two, applied by A. Abdalla [51], K. He [52], and M. Hussain [45], largely unfroze the feature cases of the network layers so that transfer learning could be performed, despite the fine-tuning of the feature parameters of each layer, converting to the case where the TPI condition was equivalent to TP = 0. In addition, there were transfer learning methods discussed in the structure of each network block; for example, N. Best [46] and W. Wu [47] used method three, which was equivalent to comparing the results obtained using TaE for some columns of the TP equivariant series when converting to TPI space. Although method one may have good potential in respect to the data set size required, we hold the view that this method was suitable for optimization between simple tasks or very similar tasks, as in the curve of TP = 0 in Figure 6. For method two, the expected target could achieve better in accuracy of the task, but this method must perfect most feature parameters in the whole network, and, according to our discussion in the TPI condition, the size of the optimization and the required data set were more related to the complexity of the task, and the time and data cost consumed were higher when the task deviation was large. According to the discussion in our TPI condition, the size of the possible optimized feature size and the required dataset were more related to the complexity of the task, and the time and data cost consumed were higher when the task deviation was larger. From the realized results, method three appeared to have the effect of the MTPI method, or even exceeded the TDS of the MPTI condition during the TaE process. However, method three was more randomized and unplanned, which required more manual experience or more efficient experimental designs (e.g., waypoints of TaE). The TaE process might consume significant time and manual labeling costs. Due to the complexity of the data and network differences, it was difficult to compare the actual values of methods one, two, and three with the results of this paper directly, but comparing the results of different TPs in Figure 7 showed that the MTPI conditions proposed in this paper had evident advantages in terms of comprehensiveness and systematic planning.

## 3. Materials

### 3.1. Data Collection Environment and UAV Equipment

**Figure 10:**
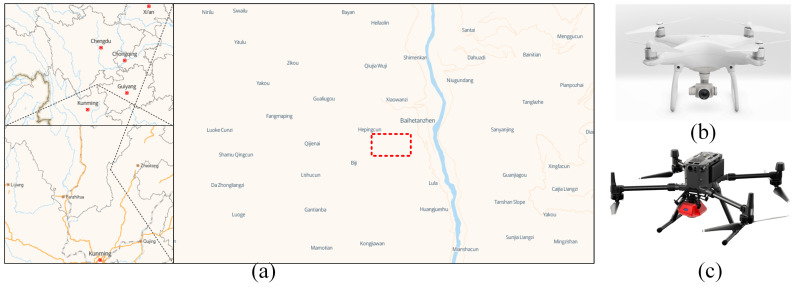
Locations of data collected sites and UAVs (unmanned aerial vehicles) equipment for images acquisition. (**a**). The study site on the map. (**b**). The DJI Phantom 4 v2 UAV. (**c**). The DJI Matrice 300 UAV. The Red dotted means the extent of the data collection site in the map.

The study site was conducted in a typical DTV climate area in Qiao-jia County, Zhaotong City, Yunnan Province, China (102°53′10.83′′ E (east), 27°10′8.96′′ N (North)), shown in Figure 10a. The annual evapotranspiration (about 2400 mm) in this region is about 3 times higher than the annual rainfall (800 mm) [53], and the rainfall during the rainy season (from about June to October each year) accounts for approximately 90% [54], with significant vegetation differences between rainy and dry seasons. The image data were collected on two randomly selected rectangular areas (approximately 500 × 750 m^2^) on the shady and sunny slopes of the same hillside. The area receives sufficient moisture, and the evapotranspiration was similar in the rainy season, so the vegetation of the two slopes is very close to each other. However, during the dry season, the evapotranspiration is higher on the sunny slopes than on the shady slopes, which makes the vegetation image features of the two slopes different.

To acquire images of vegetation distribution on shady and sunny slopes in the DTV in the rainy and dry seasons, two consumer-grade UAVs (unmanned aerial vehicles) were used to collect high-resolution low-altitude image data, including DJI Phantom 4 v2 and DJI Matrice 300 (DJI, DJI Innovation Technology Co., Ltd., Shenzhen, China) as shown in Figure 10b,c. The main parameters of the two UAVCSs (UAV-camera systems) are listed in Table 3, including positioning accuracy, UAV parameters, and camera parameters (RGB camera, RGB means Red/Green/Blue). Furthermore, the controllers of the two UAVCSs were Android smartphones (HUAWEI P20 PRO, Huawei, Shenzhen, China) + DJI pilot apps (DJI, Shenzhen, China)) with software control + manual remote control (Emergency). In addition, the Phantom 4 v2 UAV was controlled by the Android smartphone + DJI Pilot App + manual remote control (when in an emergency), whereas the DJI Matrice 300 UAV uses a DJI remote control with screen industry edition remote control with an integrated 5.5-inch 1080p high-brightness display.

### 3.2. Data Collection and Dataset

**Figure 11:**
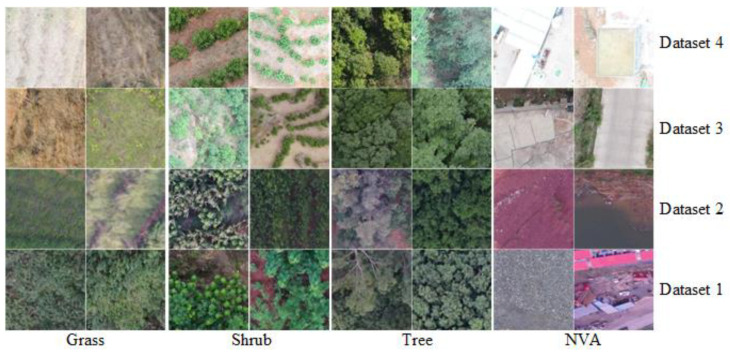
Several examples of grass, shrubs, trees, and NVA in four datasets ($D_{n}$) (size of 400^2^ (400 × 400) pixels, NVA means non-vegetation area).

The four data components (${data}_{n}$, n=1,2,3,4) were collected in two batches during dry and rainy seasons: n=1 meant shady in rainy (means shady slope image in and the rainy season), n=2 for sunny in rainy, n=3 for shady in dry, and n=4 for sunny in dry. The ${data}_{1,2}$ were collected in mid-August 2021 (the middle of the rainy season, when the rainfall is almost the heaviest), and the ${data}_{3,4}$ were collected in early June 2021 (the end of the dry season, when vegetation is affected by the dry season for a longer period). During data acquisition, the UAVCSs were flown at an altitude of 200 m (vertical height relative to the center of the area), so the image resolution was about 5 px/cm. The heading overlap rate was set to 70% and 75% for lateral (with image overlap rate of 52.5% (70% × 75%)), where the heading overlap rate meant the image overlap rate parallel to the UAV direction, and the lateral overlap rate meant the image overlap rate perpendicular to the heading overlap rate. The images number of each ${data}_{n}(n=1,2,3,4)$ was 500, which could be expressed as ${data}_{n}={\left\{{IM}_{I}\right\}}_{n};\;(n=1,2,3,4;\;I=1,2,3,\ldots ,\;500)$, so the total image data collected in study site was 2000 (500 × 4). The image data ${IM}_{I}$ can be denoted as ${IM}_{I}=H\times W\times 3$, ${{IM}_{I}\;\in \;data}_{n}$; among them, the H was the image high (pixels) of the RGB image (3 channels), and the W was the image width (pixels), whereas H=3000, W=4000 (for the Phantom 4 v2 UAVCS) or H=4800, W=6400 (for the Matrice 300 UAVCS). To make hardware memory allocation and high operational efficiency proper in deep learning training and testing, the data ${\left\{{IM}_{I}\right\}}_{n}\;$were cropped into smaller localized image samples, denoted as $D_{n}={\left\{{im}_{i}\right\}}_{n};\;n=1,\ldots ,4;\;i=1,2,\ldots ,M$; while ${im}_{i}=h\times w\times 3$. The sample size of ${im}_{i}$ was unified as h=w=400 in $D_{n}$, referring to the input size (h=w=224, the build dataset and networks layers should be larger than/equal to 224) of the deep learning network established by K. He [52]. Thus, each dataset $D_{n},\;n=1,\ldots ,4$ contained 2500 image samples (M = 2500), and each sample ${im}_{i}$ was cropped in random positions of ${IM}_{I}$ (I was random integer from 1 to N ), such as several examples shown in Figure 11. Additionally, each image sample ${im}_{i}$ in the $D_{n}$ was manually labeled with corresponding segmentation label $L_{n}={\left\{{label}_{i}\right\}}_{n};\;n=1,\ldots ,4;\;i=1,2,\ldots ,M$. Additionally, each image sample ${im}_{i}$ in the $D_{n}$ was manually labeled its corresponding segmentation label $L_{n}=\left\{{label}_{i}\right\};\;n=1,\ldots ,4;\;i=1,2,\ldots ,M$. The manually labelled results included four vegetation states, for example, NVA (Non vegetated area): (magenta (255,0,255), (RGB = [0,255,255] (red/green/blue channel values))), grass (red, (255,0,0)), shrubs (green, (0,255,0)), and trees (blue, (0,0,255)).

**Figure 12:**
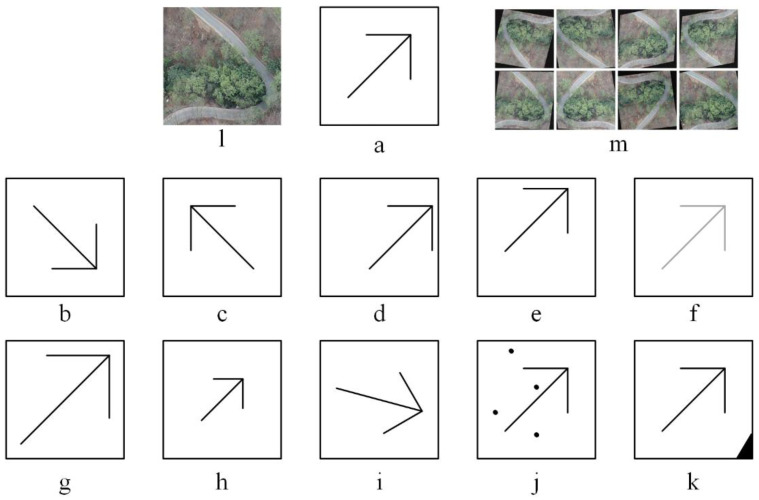
Schematic diagram of the data augmented method. (**a**). A diagram of original image sample. (**b**). Y-direction symmetric. (**c**). X-direction symmetric. (**d**). X-direction panning. (**e**). Y-direction panning. (**f**). The color degree/saturation/brightness random enhancement/reduction. (**g**). Image scaling up. (**h**). Image scaling down. (**i**). Random rotation. (**j**). Random noise. (**k**). Random cropping. (**l**). A random example (${im}_{i}$) on $D_{n}$. (**m**). Several augmentation results of (l).

To improve the model’s accuracy and generalization capability, each established dataset $D_{n}$ was augmented with 7 methods: symmetry, panning, HSV (Hue/Saturation/Value) random enhancement/reduction, scale up/down, rotation, noise addition, and cropping, as shown in Figure 12. Among them, symmetry included (random) x-direction (longitudinal) and y-direction (transverse), with the symmetry axis passing through the sample center. The random panning method included x and y directional panning from −5 to 5% (5% × h/w = 20). The random HSV enhancement/reduction was obtained by converting the RGB to HSV color space and randomly enhancing/reducing the three channels separately, with the range of 0.9~1.1. The random rotation range was ±5°, and the rotation center is the image center. The random noises used were Gaussian image noises with a mean of 0 and a variance of 0.1. The cropping process was performed only with the image part beyond the original range, so the size after data augmentation was unchanged (h=w=400). After the above seven combination methods, each dataset $D_{n}$ was augmented to 15,000 (${DA}_{n},\;{DA}_{n}={\{ia}_{i}\}$), as n=1,…,4; i=1,2,3,…, MA (MA=15,000), and the total size of four augmented datasets was 60,000 (15,000 × 4).

### 3.3. The Four Deep Segmentation Networks and Evaluation Metrics

**Figure 13:**
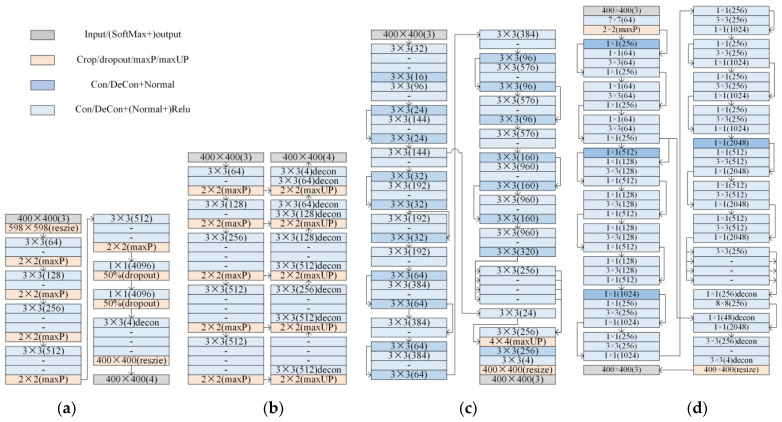
The four deep learning networks for vegetation segmentation. (**a**). The structure of FCN. (**b**). The structure of Seg-net. (**c**). The structure of Mobile-Net v2. (**d**). The structure of Res-net 50. In the figure, the size of each network layer in the networks was labeled as N × N(F), meaning the size (N × N) and the number of filters (F). In addition, the “Input/(SoftMax+) output” means the input layer or the output layer, where a SoftMax layer might omitted before the output layer. The “Crop/dropout/maxP/maxUP” means the cropping layer resize layer, dropout layer, maximum pooling layer, or maximum non-pooling layer. The “Con/DeCon+Normal” is the convolution layer + normalization layer or the convolution layer. The “Con/DeCon+(Normal+) Relu” is the convolution layer + normalization layer + relu activation layers or the deconvolution layer. The “-” represents a structure identical to its upper edge.

In this study, the environment for building, training, optimizing, and evaluating the deep learning networks was MATLAB 2023a running on a Win 11 PC with a 12G NVIDIA GeForce RTX 3060 graphics card (12,000 MHz), a 12th generation Intel(R) Core (TM) i5-12600KF CPU (3.6 GHz), and 64G DDR4 RAM (3600 MHz). The four popular deep learning network structures used in this work, including Seg-Net [52,55], FCN [56], Mobile-Net v2 [57], and Res-Net 50 [58], have been given in Figure 13. Among them, the Seg-Net is a convolutional neural network framework using an encoder–decoder structure with high resolution and detail retention when processing large-scale images or increasing the depth of the network. The FCN is a semantic segmentation model with a full convolution structure, which is fast and occupies less memory, but it may reduce resolution. The Mobile-Net v2 maintains accuracy, with a low complexity network, fast training, and good operability, but may have low accuracy for small objects. The Res-Net 50 is a segmentation network using residual blocks, which has high accuracy and few parameters, but gradient explosion may occur in the training process. The segmentation performances of the four networks were built and trained, with the most suitable deep learning network selected for segmentation and further optimized to obtain efficient results.

Five parameters were used to evaluate the performances of the four deep learning networks, including WPA (weighted pixel accuracy), WRE (weighted pixel recall), WF1 (weighted F1 score), WIoU (weighted intersection over union), and WKs (W-kappa score, weighted Cohen kappa coefficient score). Among them, WPA characterized the correctness effect of pixel segmentation, as shown in Equation (1).

(1) $$WPA=\sum_{i=1}^{C}\left({p_{i}\times precision}_{i}\right)=1/C\sum_{i=1}^{C}{TPP}_{i}/({TPP}_{i}+{FPP}_{i})$$

where, i is the type of vegetation, i=1,…,c; c=4, $p_{i}$ is the probability that the i-th-planted pixel accounts for in the overall dataset images in Equation (2), ${TPP}_{i}$ is the number of true positive pixels, ${TNP}_{i}$ is the number of true negative pixels, ${FPP}_{i}$ is the number of false positive pixels, and ${FNP}_{i}$ is the number of false negative pixels.

(2) $$p_{i}=\sum_{i=1}^{M}{\left|\left\{C_{c}\right\}\right|}_{\left(1,i\right)}/\sum_{c=1}^{C}\sum_{i=1}^{M}{\left|\left\{C_{c}\right\}\right|}_{\left(1,i\right)}$$

when$\;I_{i}$ is the pixel of the label of a dataset (for example DA), $\left|\left\{C_{c}\right\}\right|$ means the total number of pixel eligible for x. $I_{i}$ means the pixels were labeled as planted i, i=1,…,C. The WRE characterizes the ability of the model to find all positive pixels, as Equation (3).

(3) $$WRE=1/C\sum_{i=1}^{C}\left(p_{i}\times {recall}_{i}\right)=1/C\sum_{i=1}^{C}{TPP}_{i}/({TPP}_{i}+{TNP}_{i})$$

The WF1 is the harmonic mean of precision and recall (0≤WF1≤1), with higher values showing better model performance. When the accuracy and recall of the model are both high, the WF1 achieves a maximum value of 1. Therefore, the WF1 is a metric that combines the accuracy and recall of the model and can be used to evaluate the overall performance of the model, expressed as Equation (4).

(4) $$WF1=\sum_{i=1}^{C}\left(p_{i}\times (2\times {precision}_{i}\times {recall}_{i})/({precision}_{i}+{recall}_{i})\right)$$

where the WIoU is the weight IoU metric, which measures the overlap between the predicted and ground truth masks, as in Equation (5).

(5) $$WIoU=\sum_{i=1}^{C}p_{i}\times {TPP}_{i}/({TPP}_{i}+{FPP}_{i}+{FNP}_{i})$$

Finally, WKs is the kappa coefficient that represents the overall classification accuracy, which is used to parse the accuracy of the model for different frames, expressed as Equation (6).

(6) $$WKs=(P_{o}-P_{e})/(1-P_{o})$$

where $p_{e}$ is the expected probability of agreement by chance, the $p_{o}$ refers to the output sum of real classes and the number of results of the sample WKs calculates as −1 to 1, but usually kappa falls between 0 and 1, which can be divided into five groups to represent different levels of consistency: 0.0 to 0.20 extremely low consistency (slight), 0.21 to 0.40 general consistency (fair), 0.41 to 0.60 moderate consistency (moderate), 0.61 to 0.80 height of consistency (substantial) and 0.81 to 1 almost perfect.

## 4. Methods

**Figure 14:**
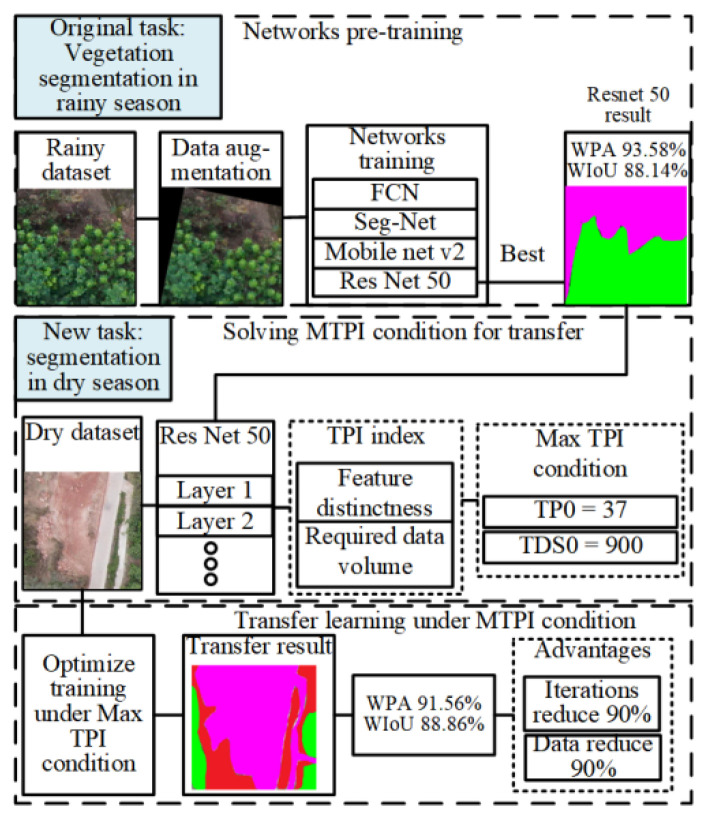
Technical flowchart of the MTPI transfer method. Blue, green, red and magenta indicate trees, shrubs, grasses and non-vegetation area in the segmentation results.

The three main phases introduced in this study included deep networks pre-training, MTPI condition solving, and pre-training network transfer learning, as shown in Figure 14.

### 4.1. Deep Learning Networks Pre-Trained

Before pre-training deep networks, the established ${DA}_{1}$ was split randomly into three subsets of ${TraningSet}_{1}$, ${ValidationSet}_{1}$, and ${TestSet}_{1}$ (4:1:1). Among them, the ${TraningSet}_{1}$ was used to pre-train the network parameters such as weights and biases. The$\;{ValidationSet}_{1}$ performed periodic tests (e.g., 100) on the intermediate and final networks obtained during the training process. The ${TestSet}_{1}$ was used to evaluate the performance and segmentation capabilities of the four networks. Then, the four deep networks were built for pre-training, including Seg-Net, FCN, Mobile-Net v2, and Res-net 50 (introduced in Section 3.3). As the field UAV image dataset possibly created imbalanced sample categories, the training process may have been biased towards large categories and reduce the segmentation performance. To reduce the problem of imbalanced categories’ training and to improve the network’s accuracy, all of the four deep networks were processed with the categories weighting method. Suppose $L_{1}^{\prime }$ was the images label set in the ${TraningSet}_{1}$, and ${label}_{\left(i,1\right)}\in L_{1}^{\prime }$ was the i−th label image. The set ${\left\{C\right\}}_{\left(i,1\right)},\;C=1,2,3,4$ was used to represent all the pixel points of each type of vegetation in ${label}_{\left(i,1\right)}$, when c=1 meant the trees pixels; c=2 meant shrubs, c=3 meant grass, and c=4 meant NVA. Then, the requested weights could be obtained using Equation (7).

(7) $$w_{c}=\frac{1}{\sum_{i=1}^{M}{\left|\left\{C\right\}\right|}_{\left(i,1\right)}};\;i=1,2,\ldots ,M$$

where |A| means cardinality of set A, and $M=|L_{1}^{\prime }|$.

### 4.2. The MTPI Method for the Pre-Trained Network

To improve the controllability and systematicity of transfer learning, the TPI was proposed to evaluate the transfer learning conditions. As TPI was evaluation index before transfer training, the hardware- and time-consuming TaE process could be avoided, whereas controllability and systematicity of a transfer learning were also improved. So, transfer learning under the condition with maximum TPI (the MTPI method) made the transfer training process more reasonable in conditions of training time and new data quantity.

To implement the TPI in terms of both training speed and final accuracy, Equation (8) was defined to represent it.

(8) $$TPI(x)={TPI}_{WKs}(x)/{TPI}_{TP}(x)$$

where x = 1, 2, 3, … is the transfer depth (TP) input, x=0 means no network layers was frozen, and x=−1 means all layers are frozen. Additionally, the x (TP, transfer point) stands for the frozen layer number in transfer training (counting from the input layer), and a layer refers to the activation layer, e.g., relu. ${TPI}_{WKs}(x)$ denotes the evaluation of the final accuracy potential, and ${TPI}_{TP}\left(x\right)$ is the potential evaluation from training time cost. As the value domain of ${TPI}_{WKs}(x)$ is from 0 to 1 (0 to 100%), and the value domain of time consumption (number of iterations/level) is greater than 1, the value range of TPI is from 0 to 1. Equation (8) also shows that the better the final precision and the lower the time cost, the larger the TPI is.

As the training time consumption in deep learning is related to the size of the new dataset (TDS, transfer dataset size) used, the TDS needs to be decided according to the feature quantity requiring optimization. Therefore, ${TPI}_{TP}$ (TPI from TP perspective) was used to respect represent a evaluate of a transfer learning condition, which was expressed as Equation (9).

(9) $${TPI}_{TP}(x)=[(\frac{LS\left(0\right)-LS\left(x\right)}{LS\left(0\right)})\times N_{level}]+1$$

where $LS\left(x\right)$ refers to the size of the partly network layer (number of parameters) that will be perfected. Moreover, the $N_{level}$ is the evaluation classification level, e.g., $N_{level}=10$. [] is the rounding function. The value domain of ${TPI}_{LP}(TP)$ is $\left\{1,2,...,N_{level}\right\}$.

To determine the TDS, the influences of the structural complexity, task complexity, and data quality of the network were ignored, and the TDS was considered to have a linear relationship with $LS\left(x\right)$, expressed as Equation (10).

(10) $$TDS(x)=k_{t}\times LS\left(x\right)$$

where $k_{t}$ is the scale factor. The TDS and LS were demonstrated to effectively train with good accuracy in the pre-training, Equation (11).

(11) $${TDS}_{pre}=k_{pre}\times {LS}_{pre}$$

where $k_{pre}$ means the coefficients assuming a linear relationship between ${TDS}_{pre}$ and ${LS}_{pre}$, the ${TDS}_{pre}$ is the dataset size in the network’s pre-training, and ${LS}_{pre}=LS(0)$ is the network’s size. Compared to the pre-training process, factors such as task difficulty in transfer training have been drastically reduced, so $k_{t}\ll k$ might be an ideal relationship, e.g., the TDS on Y.J. Wang [44], K. He [52], and A. Abdalla [51]. However, in this paper, we consider that $k_{pre}=k_{t}={TDS}_{pre}/{LS}_{pre}$ is the lowest error risk condition for transfer training.

In addition, accuracy is also a key factor to evaluate transfer training, so we consider whether the required transfer features have good classification characteristics. As the WKs can evaluate the combined classification results on multiple classifications, the WKs introduced above were also used in the evaluation of transfer potential. The potential index ${TPI}_{WKs}\left(x\right)$ (TPI from KWs perspective), which is evaluated from the accuracy perspective, is when the TP is x, which can be expressed as Equation (12).

(12) $${TPI}_{WKs}\;\left(x\right)=WKs({DA}_{n},x)$$

where ${DA}_{n}$ refers to the dataset used for transfer learning. The $WKs({DA}_{n},x)$ refers to the WKs result calculated by Equation (6) on ${DA}_{n}$. The ${TPI}_{WKs}\left(x\right)$ was expressed as the performance of the transfer training task when the transfer training set was ${DA}_{n}$, with a value range of 0 to 1 (×100%). The larger the ${TPI}_{WKs}(x)$ was, the easier to receive a better segmentation effect.

Based on Equations (8)–(10), TPI(x) index described the transferable potential of a deep network on a dataset when the TP was x. So, the MTPI method (maximum TPI index method) was proposed by solving the TPI index values of all layers in the pre-trained network, and the ${TP}_{0}$ corresponding to the maximum TPI value was the most suitable TP, expressed as Equation (13).

(13) $${TP}_{0}=arg(max(TPI))$$

In this study, before improving the pre-trained network through transfer training in a DTV environment, the combined performances of the pre-trained network in new datasets (${DA}_{2,\;3,\;4}$) were first measured. For datasets that kept outputting well in the pre-trained network (e.g., WKs greater than 0.8), we did not process them any further, and only the datasets with mediocre performance (e.g., WKs less than 0.6 in the evaluation) could be perfected by transferring training. Thereafter, the TPI curves (${TPIC}_{1,2,3,4}$) for ${DA}_{1,2,3,4}$ were obtained by combining Equations (9)–(13) (the ${TPIC}_{0}$ is the average of ${TPIC}_{1,2,3,4}$), and the MTPI conditions under the DTV data set were obtained (${TDS}_{0}$ and ${TP}_{0}$).

### 4.3. The Pre-Trained Networks Transfer to the Dry Season Dataset on MTPI Condition

To verify the effectiveness of the TPI parameter assessment for a transfer learning process, the MTPI conditions (${TP}_{0}$ and $\;{TDS}_{0}$) derived in Section 4.2 were compared and evaluated with the dataset and pre-trained network presented in this work. For the TP differences, the training accuracy and training loss trends under fixed transfer learning dataset sizes of ${TDS}_{0}$ and variations in TP (TP = ${TP}_{0}$, ${TP}_{0}$ ± 1, ${TP}_{0}$ ± 5, ${TP}_{0}$ ± 10, 0, −1) were examined. For the TDS aspect, the training accuracy and training loss variation trends were outputted with a fixed TP of TP0 and a varying size of TDS (e.g., TDS = 10, 20, 50, 100, 200, 500, 1000 (${TDS}_{0}$), 2000).

To compare the optimization effects of deep networks after the MTPI method of pre-trained networks in DTV conditions, the network outputs after transfer training were performed in MTPI conditions. The results included segmentation mapping of random samples in the ${DA}_{3,4}$. In addition, the overall statistical results (calculated by Equations (1)–(6)) and confusion matrixes of the datasets were also extracted to facilitate the comparison of the final segmentation results for each vegetation category.

## 5. Conclusions

This paper proposed an evaluation MTPI method for the transfer learning in both data quantity and the feature quantity requiring optimization, which was tested and validated in DTV environments. The main conclusions were as follows.

1. Four deep learning networks (Seg-net, FCN, Mobile-Net v2, and Res-Net 50) were trained and compared, where Res-Net 50 was found to have the best segmentation results with 93.58% accuracy and 88.14% IoU, which was selected for further transfer learning.
2. To solve the best transfer learning condition, each layer TPI of the pre-trained network in the four datasets was obtained, and the MTPI condition (${TDS}_{0}\;$ = 1000 and ${TP}_{0}$ = 37) was decided.
3. The results of comparing transfer learning with varying TDS and TP showed that the obtained MTPI condition performed best (accuracy of 91.56%, and IoU of 84.86% under a 90% reduction in the dataset and a 90% reduction in the iterations) in terms of dataset consumption and time consumption.

The proposed MTPI approach was informative when applying deep learning to transfer in complex similar scenarios, such as unstructured agriculture or geographic environments. Although the proposed method showed acceptable performance for vegetation segmentation, it might need continuing research in two areas (which will probably be in our following research). Firstly, the MTPI method considers the amount of data and features between pre-training and transfer training as equal linear relationships (although the results are acceptable), but the relationship could be much more complex. In addition, the transfer learning process of task complexity, data complexity, and model complexity also affects the TPI in this paper.

## Figures and Tables

**Table 1:** Segmentation results of the four deep learning deep networks in ${DA}_{1}$.

Deep Networks	WPA	WRE	WF1	WKs	WIoU
Seg-Net	0.7122	0.6984	0.7044	0.5859	0.5816
FCN	0.7364	0.7451	0.7277	0.6244	0.6117
Mobile-Net v2	0.8433	0.8397	0.8426	0.7701	0.7390
**Res-net-50**	**0.9358**	**0.9309**	**0.9358**	**0.9355**	**0.8814**

**Table 2:** Segmentation results of pre-trained network (Res-net 50) on datasets ${DA}_{2,\;3,\;4}$.

Test Dataset	WPA	WRE	WF1	WKs	WIoU
${DA}_{2}$	0.9352	0.9288	0.9353	0.9032	0.8803
${DA}_{3}$	0.4382	0.4443	0.4833	0.2407	0.2529
${DA}_{4}$	0.1705	0.3464	0.2367	0.0094	0.0984

**Table 3:** The parameters of UAVs for data acquisition.

UAV	Matrice 300	Phantom 4 v2
Lens FOV	45°	84°
Equivalent focal length	25 mm	35 mm
Positioning mode	GPS + RTK	GPS/GLONASS
Vertical positioning accuracy	±0.1 m	±0.1 m
Horizontal positioning accuracy	±0.1 m	±0.3 m
Image acquisition mode	no hover shooting	no hover shooting
Resolution of location	Less than 50 mm	Less than 50 mm
Flight altitude	<500 m	<500 m
Flight speed	5 m/s	6 m/s
Aperture	Aps-C	f/2.8–f/11
Camera model	FC2008	FCS400
Shutter	1/2000	1/2000
Resolution	3000 × 4000	4800 × 6400
ISO range	100–6400	100–12,800
Photo format	JPEG/RAW	JPEG/DNG

## Data Availability

Data are available from the first authors upon request.

## Nomenclature

Symbols	
DTV	Dry Thermal Valley
FCN	Fully Convolutional Networks
GAN	Generative Adversarial Networks
HSV	Hue/Saturation/Value
IoU	Intersection over Union
LD	Layer depths
LSTM	Long Short-Term Memory
MTPI	Maximum Transfer Potential Index method
NVA	Non vegetated area
Res-Net 50	Residual Network
RGB	Red/Green/Blue
Seg-Net	Semantic Segmentation Networks
TaE	Trial-and-error method
TDS	Transfer dataset size
TP	Transfer point/transfer depth
TPI	Transfer Potential Index
UAV(s)	Unmanned aerial vehicle (s)
UAVCSs	UAV-camera systems
WF1	Weighted F1 score
WKs	W-kappa score, weighted Cohen kappa score
WIoU	Weighted IoU
WPA	Weighted pixel accuracy
WRE	Weighted pixel recall
